# Maryland Power Plant Cooling-Water Intake Regulations and their Application in Evaluation of Adverse Environmental Impact

**DOI:** 10.1100/tsw.2002.175

**Published:** 2002-03-08

**Authors:** Richard McLean, William A. Richkus, Stephen P. Schreiner, David Fluke

**Affiliations:** Power Plant Research Program, Mmaryland Department of Natural Resources, Annapolis, MD 21401, USA; Versar, Inc., Columbia, MD 21045, USA; Maryland Department of Environment, Baltimore, MD 21224 , USA

**Keywords:** entrainment, impingement, environmental impact, cooling water regulation

## Abstract

Maryland’s cooling-water intake and discharge regulations, the Code of Maryland Regulations (COMAR) 26.08.03, stem from Sections 316(a) and (b) of the Clean Water Act (CWA). COMAR 26.08.03.05 and litigative and administrative rulings stipulate that the location, design, construction, and capability of cooling-water intake structures must reflect the best technology available (BTA) for minimizing adverse environmental impacts (AEIs), providing that the costs of implementing the BTA are not wholly disproportionate to the expected environmental benefits. Maryland law exempts facilities that withdraw less than 10 million gallons/day (MGD) and less than 20% of stream or net flow by the intake. If not exempt, BTA must be installed if the cost of doing so is less than five times the value of fish impinged annually. Through site-specific studies and the use of a Spawning and Nursery Area of Consequence (SNAC) model applied to Representative Important Species, several power plants were evaluated to determine if they have had an adverse effect on spawning and nursery areas of consequence. Examples of application of the Maryland law to a number of power plants in the state are presented, together with the outcome of their evaluation.

## INTRODUCTION

Maryland takes pride in its strong commitment to environmental protection. A cornerstone of this commitment has been the state's efforts to restore and protect the

Chesapeake Bay and all of its diverse natural resources. One of the initial steps toward protecting the bay was the creation in the early 1970s of the Power Plant Research Program (PPRP). PPRP was created by legislation in response to public controversy that arose when the Baltimore Gas and Electric Company (BG&E) announced plans to construct the large Calvert Cliffs Nuclear Power Plant along the shoreline of the bay. This plant would withdraw large volumes of cooling water from the bay and discharge the heated water back into bay waters. The public was concerned about the potential for the plant to adversely affect the bay and its fisheries resources, and the state could not respond to these concerns because it did not have adequate technical expertise with regard to the potential impact that these power generating facilities might have on the bay.

As a result, the legislature created PPRP to ensure that, in the future, all existing and proposed power generating and transmission facilities in Maryland would operate in a manner that ensured protection of the state's natural resources and at the same time made electric power available to the public at reasonable rates. With regard to proposed new generating and transmission facilities, PPRP is charged with assessing and advising the Maryland Public Service Commission on the environmental and economic considerations associated with the siting, design, and operation of the proposed facilities. For existing facilities, PPRP provides technical assistance in permit review and evaluation to the Maryland Department of Environment (MDE), which is the state's permitting agency with responsibility for writing national pollution discharge elimination system (NPDES) permits and enforcing compliance with permit provisions.

Since its inception, PPRP has provided technical reviews of issues and developed recommendations concerning requirements associated with Maryland's regulations for cooling-water intake structures (CWIS) for all its generating stations. PPRP works cooperatively with MDE in reviewing all data and information required from plant operators by MDE. In many instances, the state has conducted research independent of permittees in order to assess impacts and technologies to reduce those impacts. The information presented in this paper is based on PPRP's experience in addressing CWIS issues and on the results of the program's very diverse yet comprehensive studies of the manner in which cooling-water withdrawals have impacted aquatic biota in Maryland's waters.

## MARYLAND REGULATIONS FOR CWIS

### Impingement

As generating stations draw water in for the cooling cycle, aquatic organisms near the intake are caught in the suction and fatally trapped (impinged) on the intake screens. The best technology available (BTA) for impingement was deemed by Maryland to be the technology that was the most cost effective for reducing the magnitude of impingement impact, as established by the value of the fish lost to impingement. Thus, as established in the Code of Maryland Regulations (COMAR) 26.08.03.05.D(1) and D(2), the dollar value of the organisms killed by impingement is to be calculated, and the plant operator is required to implement technologies to reduce impingement only to the extent that the cost to the facility does not exceed the total value of lost organisms over a 5-year period (in practice, generally five times the value of fish lost to impingement in a single year). In essence, Maryland's BTA is based on a simplified cost-benefit assessment.

The technical basis for the regulation is not documented in the state's regulatory records. We believe that the dollar values of fish presented in Section 08.02.09.01 of COMAR were taken from the American Fisheries Society's (AFS) listing of fish values at the time the regulation was promulgated. AFS has regularly updated its fish values, and those values are used throughout the country to establish the costs of fish kills due to many causes. Maryland's regulation did not specify changes of those values over time (for example, to account for inflation or devaluation). Thus, the values in Section 08.02.09.01 have not been modified since they were first promulgated. Plants using cooling water in the state have been evaluated under these regulations since the 1970s. MDE and DNR reviewed the issue of static fish values in their assessments throughout the period.

## Entrainment

Aquatic organisms that are drawn through the generating facility through cooling systems, intake valves, and turbines may be injured or killed as they are pulled (entrained) through the station. The general concepts underlying a determination of BTA for entrainment by Maryland are as follows:

- The evaluation of impact should be carried out to a specified level of biological significance, i.e., representative important species (RIS) and spawning and nursery areas of consequence for the RIS.
- The consequences of the cooling-water withdrawal effects should be based on the extent to which they impact the viability of the RIS population and the ecosystem necessary to support its life history functions. The effect of the cooling-water intake itself (i.e., the number of fish impinged or entrained) should not be the major focus; it is the consequence of that effect to the biological entity of concern, whether at the species or the ecosystem level, which establishes what actions the state will take.

The state determined that a sequential approach to entrainment impact assessment is a good, generic approach to the issues involved, with the steps in that sequence being (1) to quantify the effects of the cooling-water withdrawal (i.e., estimate the numbers of organisms lost to entrainment), (2) to establish the biological entity at risk (i.e., select RIS), and (3) to assess the significance of the effects for causing adverse harm to the target entity.

The CWIS operator is required to determine if the entrainment loss results in a significant adverse environmental impact (AEI), which is defined as a statistically measurable effect outside the plant's mixing zone. Entrainment evaluation modeling has been applied in Maryland assessments[1,2].

## WATER WITHDRAWAL RATE THRESHOLD

Maryland regulations also establish a water withdrawal rate threshold below which impacts are assumed to be sufficiently small as to not require regulation for BTA. The state defines that threshold as 10 million gallons/day (MGD), if that volume of water is less than 20% of the defined flow for the providing water body: design stream flow (7Q10) for nontidal waters (rivers), and the annual average net flow past the point of discharge which is available for dilution for tidal waters. Note that this exemption takes into account site specificity (i.e., the size of the body from which the water is withdrawn), reinforcing the regulation's intent that facilities be evaluated on a site-specific basis. No documentation exists within Maryland's regulatory archives to explain the technical basis for the 10-MGD threshold. However, discussions with individuals involved in the development of the regulation suggested that the threshold value was selected based on knowledge of the various facilities in the state that withdrew cooling water from the state's surface waters; the status of the ecosystems from which that water was being withdrawn; and the professional judgment of the resource managers and permit regulators with management and regulatory authority at that time. Since then, the state has not modified that threshold, and no impacts have occurred that have supported the need for its reassessment.

Maryland CWIS regulations do not vary according to specific water body type except with regard to the way in which the allowable percentage withdrawal threshold is calculated. Two reasons underlie that decision. First, a site-specific assessment approach was adopted, which makes generalizations related to water body type moot. Second, a site-specific approach was established because the potential for adverse impact was not consistent within each water body type. For example, the regulation did not differentiate between estuarine and fresh waters, recognizing that not all locations within an estuary or a freshwater body are equally sensitive or productive.

## IDENTIFYING AND ADDRESSING IMPACTS

### Defining Adverse Environmental Impacts

Approaches to minimizing adverse impacts must be based on strong technical data and information. Maryland regulations do not specify the types of studies required to provide the data needed to comply with the regulations. However, because the PPRP existed at the time the regulations were put in place, utility study designs and results of studies were evaluated in a fairly consistent manner, and the state's approach to such evaluations was increasingly refined over time. Also, most of the generating stations in the state were owned by two major utilities—BG&E and Potomac Electric Power Company (PEPCo)—and the utility approach to satisfying the state's requirements became fairly standardized; the same utility staff worked with the same state agency and contractor staff for more and more facilities.

Continued or periodic monitoring is required to measure the effectiveness of a given technology's performance. If the state's CWIS determination required that a facility take some action, monitoring of the required action was made a requirement of the permit issued. The performance measures that would be used to measure BTA effectiveness were made facility- and site-specific, and a function of the type of action required. Thus, the state did not establish any type of standardized monitoring requirement related to CWIS determinations.

Quantification of the effects of water withdrawal is necessary but not sufficient to determine whether additional measures may be necessary to reduce these effects. As noted above, the key is whether the effects caused by the water withdrawal have significance to the biological entity of concern. If the effects are not significant, existing structures and operations are sufficient since there is no truly adverse impact to be minimized. Thus, clearly defining what constitutes adverse impact is crucial. Maryland considers all costs to the citizens of the state in making regulatory determinations, and factors include impacts to the state's living resources and economic costs to the utilities (and, beyond, to the consumers) of measures that could be taken to reduce the effects of water withdrawal. Maryland's regulations thus balance these considerations so that any measures required of the utilities are commensurate with the estimated significance of the effects being reduced. We believe that as 316(b) rules are developed for the nation, the U.S. Environmental Protection Agency should define AEI and place AEI into context with the costs of protecting natural resources.

## Defining Best Technology Available

Based on extensive research and data, Maryland has determined that the extent of impacts of cooling-water withdrawal is site specific, as are the need for and the nature of various ameliorating intake technologies. Factors that directly affect the decisions on what constitutes BTA at a particular facility include a determination of an impact, the nature of that impact, the design and location of the facility on the water body, and life stages of affected species. Maryland's regulations do not specify a design intake velocity; Maryland facilities generally have a 1 to 2 ft/s screen face velocity. Impingement rates at Maryland plants with similar intake designs within the Chesapeake Bay have varied widely, and they appear to be related more to the plant's location and the location of the intake than to intake velocity or volume of water withdrawn.

Our assessments of generating facilities in Maryland resulted in BTA determinations that ranged from a decision that the existing intake structure is BTA to recommending mitigative technologies such as wedgewire screens, modifications to intake structures, and installation of barrier nets. Therefore, we believe there is no single technology or suite of technologies that can be applied on a state-wide or nation-wide basis. We believe, however, that it is important to have a consistent national process for identifying BTA at the site-specific level.

## Cumulative Impacts

Cumulative effects of impingement and entrainment are not specifically addressed in the regulations, but they have been evaluated in Maryland in a limited and somewhat cursory manner. Most Maryland facilities are relatively far apart spatially, and the biological populations exposed to the effects of these widespread plants are often distinct, with only some intermingling. For example, the major tributaries of the Chesapeake Bay support their own spawning populations of striped bass (*Morone saxatilis*), and impacts to the Potomac River stock would have no significance to the Nanticoke River stock. Maryland has tracked cumulative impingement losses across all power plants for some species, such as Atlantic Menhaden (*Brevortia tyrannus*), that may occur over a wide range of salinity regimes and are thus exposed to the effects of all of the power plants located on tidal waters of the state. These assessments have suggested that the cumulative magnitude of impingement is a small fraction of the commercial harvest of the species and a small fraction of the amount of the species consumed by predators. On that basis, the state concluded that the levels of impingement by Maryland's power plants do not represent a significant adverse impact to important resource species in the bay. With regard to Maryland's experience, long-term monitoring of the status of important resource species have temporally addressed cumulative impacts. None of these diverse monitoring programs has suggested any adverse cumulative impact from the power plants operating in Maryland[3,4].

### Mitigation

While mitigation is not identified or mentioned in Maryland's regulations, out-of-kind mitigation has been incorporated into some state NPDES permits issued after a CWIS evaluation, as is discussed further below. The state believes that mitigation can play a valuable role in the resolution of 316(b) issues on a site-specific basis. The term mitigation as used here refers to actions aside from alternative intake technologies or operating strategies that might be used to minimize ultimate impacts of cooling-water intakes to the state's resources. Mitigation may include alternative measures that can indirectly compensate the public for resource losses due to CWIS effects.

## DISCUSSION

### Maryland Facilities' Regulation Compliance

Before reviewing the permitting actions at various facilities, some general observations can be made about how facility permitting often proceeded. COMAR 26.08.03.05D addresses impingement and requires a facility owner to estimate the value of fish lost to impingement over a 5-year period as a basis for determining if modification of the CWIS to achieve BTA would be required. As a result, some quantification of magnitude and composition of impinged organisms was performed at all Maryland plants at which the water withdrawal rate exceeded the 10-MGD threshold. For those facilities where impingement was anticipated or known to be low, a relatively limited sampling effort was often sufficient to confirm that judgment. Conversely, at large plants where very substantial numbers of organisms were expected or known to be impinged, impingement studies in a number of cases were conducted over many years (e.g., 21 years at Calvert Cliffs) to ensure that an accurate characterization of impingement was made[4].

COMAR 26.08.03.05E, which addresses entrainment, does not provide guidance and requirements as detailed as those specified for impingement. Also, data and information that would be required for a rigorous empirical quantification of entrainment impact was most often unavailable and frequently was costly to acquire. For these reasons, initial estimation of whether a facility impacted a Spawning or Nursery Area of Consequence (SNAC) was often done through modeling. PPRP developed a SNAC model for that purpose that was used to estimate entrainment losses of vulnerable RIS, the consequences of those losses to the ecosystem, and the economic value of those losses[1]. An overview of that model was presented by Richkus and McLean[3]. PPRP applied the SNAC model to many of the generating stations in Maryland, and decisions on permitting and permit conditions were often based on the model outcomes. In many cases, results of the SNAC model suggested that impacts were not significant and that existing CWIS could be considered to be BTA. In cases where the SNAC model results suggested that significant impact might be occurring, but where the modeling was conducted using limited data or information from the literature, permits were issued that required the facility owner to conduct studies sufficient to reliably estimate entrainment impacts. Results of such studies were then used as a basis for subsequent permitting decisions.

PPRP assessments of the type just described established that many of the power plants in Maryland were causing minimal impacts due to entrainment and impingement. For example, at the R.P. Smith plant, which is located on the mainstem of the nontidal portion of the Potomac River, annual impingement losses were valued at $90 using COMAR-specified values, and the overall projected ecological impact from entrainment was estimated at less than 0.1% of system net primary production. Small impacts were also estimated for the Dickerson plant, which is also located on the nontidal Potomac River. Similarly minor impacts were found for some of the smaller facilities located on estuarine waters (Baltimore City), such as the Baltimore Refuse Energy Systems Company (BRESCO) waste-to-energy incinerator and the Gould Street Plant, an older facility seldom run at full capacity. For these types of projects, the existing plant CWIS was determined to be BTA and no CWIS modifications or other 316(b) action by the facility owner were required in the permit.

At some facilities, initial estimates of entrainment impacts, derived from SNAC modeling, suggested that significant impacts may be occurring, but no data were available to validate those estimates and confirm the impacts. The H.A. Wagner facility on Baltimore Harbor presents an example of such a situation. At Wagner, SNAC modeling suggested that up to 49% of the local population of bay anchovy and 17% of the silverside population could be lost to entrainment. Because of the uncertainty regarding the validity of modeling results, the facility owner was required to conduct extensive ichthyoplankton studies according to a study design reviewed and approved by the state. These studies would provide the data needed to make a more rigorous impact assessment. Analysis of data from the studies suggested that impacts were not as great as the modeling suggested, and not sufficient to warrant major CWIS modification. Thus, no modification to CWIS was required in the permit for that facility.

At large facilities utilizing once-through cooling systems, such as the Chalk Point Generating Station on the tidal Patuxent River and the Calvert Cliffs Nuclear Power Plant on the mainstem Chesapeake Bay, the large volumes of cooling water withdrawn (e.g., 3,456 MGD at Calvert Cliffs) suggested a high potential for significant impacts. Extensive and comprehensive impact assessment studies were conducted at Chalk Point by PPRP and PEPCo, the owner of the facility at that time. Similarly, at Calvert Cliffs, BG&E, the facility owner at that time, was also required to conduct comprehensive studies to comply with technical specifications in their Nuclear Regulatory Commission (NRC) license for this nuclear facility. PPRP conducted many complementary studies, which were well-coordinated with the BG&E studies.

Ichthyoplankton studies at Chalk Point indicated the potential for significant losses of forage species (bay anchovy, naked goby, silversides) in the Patuxent River estuary. Such losses could adversely affect the successful completion of the life cycles of other important species that use the Patuxent as a spawning and nursery area[5]. Based on field studies, PEPCo concluded that the reduction in anchovy recruitment for the Patuxent was 4% and that entrainment mortality could cause a reduction in forage fish biomass of about 3,000 to 15,000 lb (dry weight)[6]. These estimates were based on field measurements of population size in the Patuxent and entrainment by Chalk Point. An independent analysis of the same data by PPRP indicated that loss of bay anchovy in the estuary due to entrainment might range from 14 to 51% of the population (most probably 20 to 30%) annually[7]. PEPCo calculated the value of the entrainment losses at $150,000/year (1989 dollars) based on its loss estimates. PEPCo also calculated the cost of BTA alternatives (cooling towers and wedgewire screens) as ranging from $10,000,000 to $288,000,000 (1989 dollars). According to PEPCo, the alternatives that were evaluated varied in effectiveness in reducing entrainment from almost none to 100%. As is evident, there was substantial disagreement between the state and the utility regarding the magnitude of entrainment losses and costs of various BTA alternatives.

The substantial magnitude of the scientific and economic disagreements between the parties led to the initiation of negotiations that resulted in a mitigation alternative that was agreeable to both the state and the utility. A major factor leading to the conclusion that the mitigation option was appropriate was the substantial difference between the cost of effective BTA (such as cooling towers) and the projected environmental benefits. In 1991, MDE issued to PEPCo a national pollution discharge elimination system (NPDES) permit that required PEPCo to spend $200,000/year through 1997 on aquaculture of striped bass or other species as requested by the Maryland Department of Natural Resources (DNR), and $50,000/year for aquaculture of yellow perch or other species as specified by DNR. This permit condition called for the production of 200,000 striped bass and 50,000 yellow perch per year, with those fish being used to enhance and restore stocks in the Patuxent River. The permit also required PEPCo to provide $100,000/year to the state for environmental education or for projects to remove obstructions to anadromous fish in the Patuxent River watershed. The state of Maryland believes that a sound decision was made based on the success of the mitigation program. In this case, this program included creating a fish hatchery for potentially impacted fisheries and provision of funds for removal of obstructions to migratory fishes on tributaries by removing dams or providing fish passage facilities. The hatchery and stocking program resulted in the production and release of 3.5 million juvenile striped bass to date, the total estimated weight of which exceeded the estimated weight of forage fish lost from entrainment at Chalk Point. At the end of 1997, 750,000 American shad had also been produced. This species is currently the focus of fishery restoration efforts in Maryland. Each of these benefits is directly related to the enhancement of the state's fisheries. While continuation of the aquaculture program is not mandated in the current Chalk Point, the facility owner has continued production and release of fish in cooperation with the state.

At the Calvert Cliffs Nuclear Power Plant, which is located on the mainstem of the Chesapeake Bay, nearly 2 decades of studies were conducted during the construction and initial operation of the two units that comprise the facility. Entrainment at the plant was determined not to be a major concern because the cooling water intake was not located in a spawning area of significance. SNAC model estimates of economic loss due to entrainment were $200 annually, with overall ecological loss being 0.1% of net primary productivity. Naked goby eggs and larvae made up a large proportion of the icthyoplankton entrained, primarily because this species colonized the rip-rap used to line the intake embayment, and their eggs and larvae were being released directly into the cooling-water withdrawal flow.

Impingement at Calvert Cliffs was initially substantial with the numbers of menhaden impinged in several 1975 episodic events sufficiently high to cause intake screen collapse and plant shut-down[4]. Those initial large impingement episodes were associated with low dissolved oxygen in the intake embayment, a problem resolved in part by removal of several skimmer wall panels. Monetary value of fish lost to impingement averaged less than $25,000/year as a result of the relatively high survival of many species impinged and as a result of the relatively low value of the dominant species[4], and no CWIS modifications were required in the Calvert Cliffs permit. However, over a 14-year period, BG&E optimized their intake, screening structures, and operations such that impingement losses in the early 1990s were 10 to 50% of the losses recorded in the 1970s.

## CONCLUSIONS

The overview of impact assessment results and the detailed discussions of permitting actions at different categories of generating facilities in Maryland reinforce the basis for Maryland's perspectives on AEI presented earlier in the paper:

- Quantification of the effects of water withdrawal (i.e., numbers of organisms lost due to entrainment and impingement) is necessary but not sufficient to determine whether AEIs are occurring; the key is whether these effects are of consequence to a biological entity of concern (e.g., RIS population).

- Costs to the living resources and economic costs to the utilities and, ultimately, to the consumers must be taken into account when making permit decisions.
- The extent of impact of cooling-water withdrawal should be evaluated on a site-specific basis.
- In some instances, mitigation of some type may be the best way to ensure that the public's interests are addressed when CWIS decisions are made and permits are issued, approved, and enforced.

## BIOSKETCHES

**Richard McLean** is Manager of Nuclear Programs, Power Plant Research Program, Maryland Department of Natural Resources. He holds a B.S. in Biology and has 30 years experience in power plant impact assessment and regulation. Mr. McLeans's research interests include anadromous fish restoration; power plant impact assessment; nuclear power plant regulation and monitoring; and fate of radionuclides in the environment.

**William A. Richkus** is Vice President and Operations Manager, Versar, Inc., in Columbia, Maryland. He holds a Ph.D. in Oceanography from the University of Rhode Island (1974), an M.S. in Oceanography from the University of California-San Diego Scripps Institute of Oceanography (1968), and a B.S. in Zoology from the University of Rhode Island (1966). Dr. Richkus held the positions of Assistant Professor at Trenton State College in 1972, Assistant Professor at Wilkes College in 1973, Research Scientist and Senior Scientist at Martin Marietta Corporation from 1974 to 1986, and Senior Scientist, Division Director, and Vice President of Versar, Inc. from 1987 to the present. His research interests include anadromous and catadromous fisheries biology; fisheries resource management; ecological impact assessment; and assessment of power plant impacts.
